# Using 18F-FDG PET/CT to predict programmed cell death ligand expression in non-small cell lung cancer via metabolic tumour heterogeneity

**DOI:** 10.1093/bjr/tqaf034

**Published:** 2025-02-20

**Authors:** Ruxi Chang, Liang Luo, Cong Shen, Weishan Zhang, Xiaoyi Duan

**Affiliations:** PET/CT Center, The First Affiliated Hospital of Xi’an Jiaotong University, Xi’an, Shaanxi 710061, China; PET/CT Center, The First Affiliated Hospital of Xi’an Jiaotong University, Xi’an, Shaanxi 710061, China; PET/CT Center, The First Affiliated Hospital of Xi’an Jiaotong University, Xi’an, Shaanxi 710061, China; PET/CT Center, The First Affiliated Hospital of Xi’an Jiaotong University, Xi’an, Shaanxi 710061, China; PET/CT Center, The First Affiliated Hospital of Xi’an Jiaotong University, Xi’an, Shaanxi 710061, China

**Keywords:** positron emission tomography/computed tomography, non-small cell lung cancer, PD-L1 expression, metabolic heterogeneity

## Abstract

**Objectives:**

The purpose of this study is to evaluate the effectiveness of using 18F-FDG PET/CT metabolic heterogeneity to assess the programmed cell death ligand (PD-L1) expression in primary tumours.

**Methods:**

Data from 103 non-small cell lung cancer (NSCLC) patients undergoing 18F-FDG PET/CT were collected. PD-L1 expression was verified via biopsy or surgical specimens. The coefficient of variation (COV) assessed metabolic heterogeneity of the primary tumour. ROC curves evaluated the predictive potential of metabolic metrics and defined thresholds. Logistic regression examined predictors of PD-L1 expression.

**Results:**

The study included 103 patients (mean age: 63.65 ± 9.28 years), of whom 60 were male. Sixty-four patients had positive PD-L1 expression, while 39 had negative PD-L1 expression. COV was significantly higher in the PD-L1-positive group (*Z* = −2.529, *P* = 0.011), while no significant differences were noted in other parameters between the groups (*P* > 0.05 for all). The optimal cut-off value was proposed as 28.9, with sensitivity and specificity of 46.9% (34.3%-59.8%) and 82.1% (66.5%-92.5%), respectively (AUC: 0.649 (0.549, 0.741)) which can more effectively identify PD-L1-negative patients. Other metabolic parameters are less effective than COV (AUC< 0.6). In addition, COV-defined metabolic heterogeneity outperformed other metabolic parameters in predicting PD-L1 expression (*P* = 0.049) and emerged as an independent predictor.

**Conclusion:**

Metabolic heterogeneity, described by the COV of the primary lesion, is a marker for predicting PD-L1 expression in NSCLC patients. Therefore, the COV of the primary tumour may complement conventional imaging in providing immunohistochemical information before biopsy.

**Advances in knowledge:**

COV of the primary tumour can predict PD-L1 expression, potentially complementing conventional imaging for immunohistochemical information prior to biopsy.

## Introduction

Lung cancer significantly impacts human life, and non-small cell lung cancer (NSCLC) constitutes over 85% of cases. In 2023, it led to cancer mortality, contributing to 21% of cancer-related deaths.^1^ Immune checkpoint inhibitors (ICIs) are a therapeutic approach targeting the overexpression of immune checkpoints in tumour tissues. Programmed cell death ligand (PD-L1) inhibitors serve as a key targeted treatment for NSCLC, and patients with higher PD-L1 expression often experience enhanced survival advantages.^2^ The detection of PD-L1 status in NSCLC patients often relies on biopsy, which can cause additional trauma. Due to disease progression, PD-L1 expression at the time of biopsy may not reflect treatment status, and spatial heterogeneity within the tumour may lead to mismatched expression at the biopsy site.^3^ Therefore, a non-invasive method for measuring PD-L1 status is needed to support clinical decision-making.

Imaging tests are widely utilized to investigate PD-L1 expression due to their accessibility and repeatability. However, traditional imaging struggles to capture lesion heterogeneity and biological activity. By integrating anatomical and metabolic data, 18F-FDG PET/CT provides molecular-level insights into tumour metabolism. Research has revealed that SUVmax derived from such scans is effective in predicting PD-L1 expression, especially in lung adenocarcinoma patients.^4^ However, SUVmax (Maximum of Standard uptake value) only reflects the highest uptake in the lesion and cannot capture the differences in uptake within the tumour. The histological heterogeneity of tumour tissue is closely related to tumour progression. The coefficient of variation (COV) measures metabolic heterogeneity in 18F-PET/CT by combining mean and standard deviation (SD) of lesion uptake. Research has demonstrated COV’s role as an independent predictor of lymph node metastasis in NSCLC.^5^ However, there is currently no research exploring the relationship between tumour metabolic heterogeneity and PD-L1 expression in NSCLC. We speculate that the lesion metabolic heterogeneity of NSCLC may be related to PD-L1 expression.

This study aims to assess the ability of 18F-FDG PET/CT metabolic parameters to predict PD-L1 expression in NSCLC patients and explore whether metabolic heterogeneity can inform immunohistochemical responses, supporting immunotherapy decisions.

## Methods

### Study population

**Figure 1.**
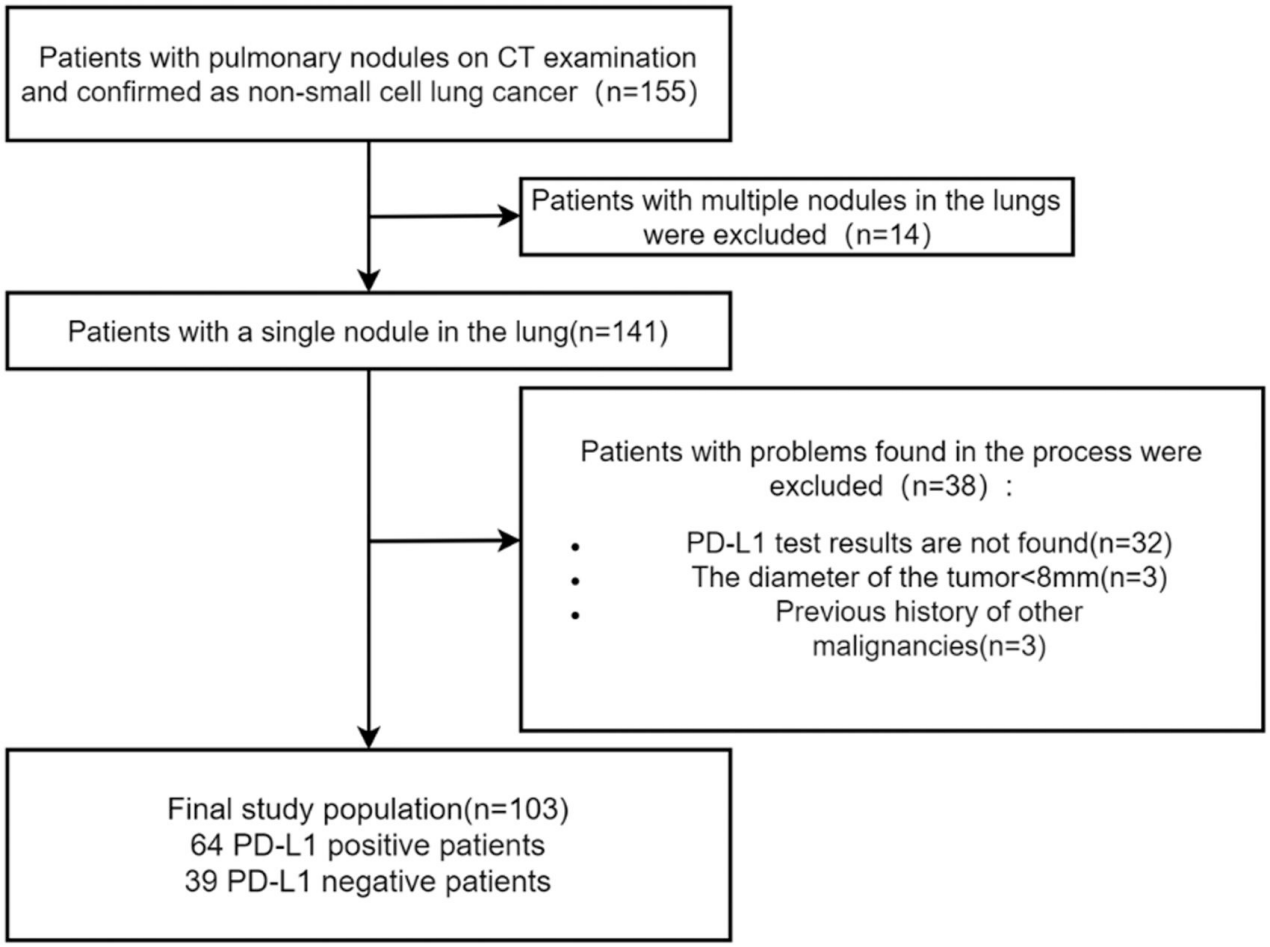
Study patient enrolment flowchart.

The study was retrospective, using data from NSCLC patients who underwent 18F-FDG PET/CT scans between January 2020 and February 2024. Inclusion criteria included: (1) confirmation of a single lesion on CT, (2) biopsy or surgical pathology confirmation with PD-L1 expression, (3) PET/CT imaging within 1 month of biopsy/surgery, with chest CT and 1 mm thin-layer reconstruction, and (4) no prior cancer or related treatments before biopsy or surgery. Exclusion criteria included: (1) tumour diameter < 0.8 cm impacting lesion labelling and (2) prior malignancy history. A flowchart of enrolment is shown in Figure 1.

### Imaging acquisition and reconstruction

Using the Philips Gemini TF PET/CT scanner (Amsterdam, Netherlands), with daily calibration of the multi-slice spiral CT and PET systems before each examination. After fasting for at least 6 h and ensuring blood glucose levels were within the normal range (<11.1 mmol/L), patients underwent 18F-FDG PET/CT scans. A dose of 3.7 MBq/kg of 18-F FDG was injected based on patient weight, and imaging was done 60 min later. The CT scan parameters included 120 V voltage, automatic current modulation (200 mAs), a slice thickness of 1 mm, and a 512 × 512 matrix. PET imaging was done with 5 mm slice thickness, 128 × 128 matrix, with 8 bed positions and 1.5 min per position. CT images were reconstructed using standard methods, and PET datasets were reconstructed using the time-of-flight method with 3 iterations. The reconstructed PET and CT images were overlaid to obtain fused images.

### Imaging analysis

PET/CT images were analysed by a nuclear medicine physician using Fusion Viewer software with a threshold of 40% SUVmax to automatically delineate 3-dimensional ROIs (Region of Interest) on the Extended Brilliance workstation. Obtain SUVmax, SUVmean, SD of SUV, metabolic tumour volume (MTV), total lesion uptake (TLU), where TLU is defined as MTV × SUVmean, COV, where COV is defined as SD/SUVmean*100.^6^ Quantitative results were measured twice by a nuclear medicine physician within a 1-month interval to ensure the reliability of the measurements.

### Statistical analysis

Data were presented as mean ± SD or percentage using GraphPad Prism 10.1.2, Python 3.9, and R software 4.2. Depending on the normality of continuous data, either independent 2-sample *t*-tests or Mann–Whitney U tests were used. The chi-square test assessed differences in clinical data and PET/CT metabolic parameters between PD-L1 positive (TPS (Tumor Proportion Score) ≥ 1%) and negative (TPS < 1%) patients. ROC curves evaluated the ability of each parameter to predict PD-L1 expression and determine cut-off values. Logistic regression was applied to analyse the effectiveness of parameters in predicting PD-L1 expression, with a significance level of *P* < 0.05.

### Patient characteristics and image characteristics

**Figure 2.**
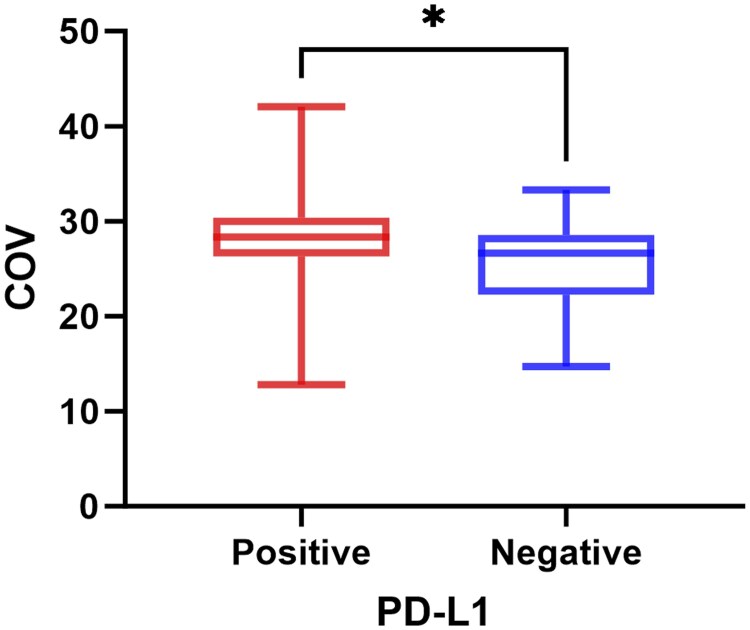
Comparison of COV between pathologically confirmed PD-L1 positive and negative group. * indicates a statistically significant difference. Abbreviations: COV = coefficient of variation; PD-L1 = programmed cell death ligand.

This study included 103 patients, as detailed in Table 1, with clinical and imaging data. Pathologically, 87 cases were adenocarcinoma, 15 squamous cell carcinoma, and 1 unclassified NSCLC. Among them, 64 were PD-L1 positive and 39 were negative. The average age of the PD-L1-positive group was 64.70 ± 9.43, and the negative group was 61.92 ± 8.87, with no significant difference (*P* = 0.141). No significant differences were found in gender, SUVmax, TLU, or MTV. However, the COV was significantly higher in the positive group (28.13 ± 5.12) than in the negative group (25.60 ± 4.86, *P* = 0.011) (Figure 2).

**Table 1.** Characteristics of patients.

	Positive (64)	Negative (39)	*t*	*Z*	χ^2^	*P*
Age, years (mean ± SD)	64.70 ± 9.43	61.92 ± 8.87	−1.484	–	–	0.141
Gender			–	–	2.346	0.126
Male	41 (64.06%)	19 (48.72%)				
Female	23 (35.94%)	20 (51.28%)				
SUVmax (median [25%, 75%])	7.20 [5.28, 10.38]	6.55 [2.80, 9.55]	–	−1.397	–	0.162
MTV	6.35 [3.98, 11.65]	5.80 [3.65, 14.39]	–	−0.105	–	0.916
TLU	23.48 [14.46, 66.30]	21.12 [5.81, 94.14]	–	−0.639	–	0.523
COV	28.36 [26.44, 30.36]	26.58 [22.49, 28.50]	–	−2.529	–	0.011*

Abbreviations: COV = coefficient of variation; MTV = metabolic tumour volume; SD = standard deviation; TLU = total lesion uptake.

* *P* < 0.05.

### Comparison between COV and other metabolic parameters

**Figure 3.**
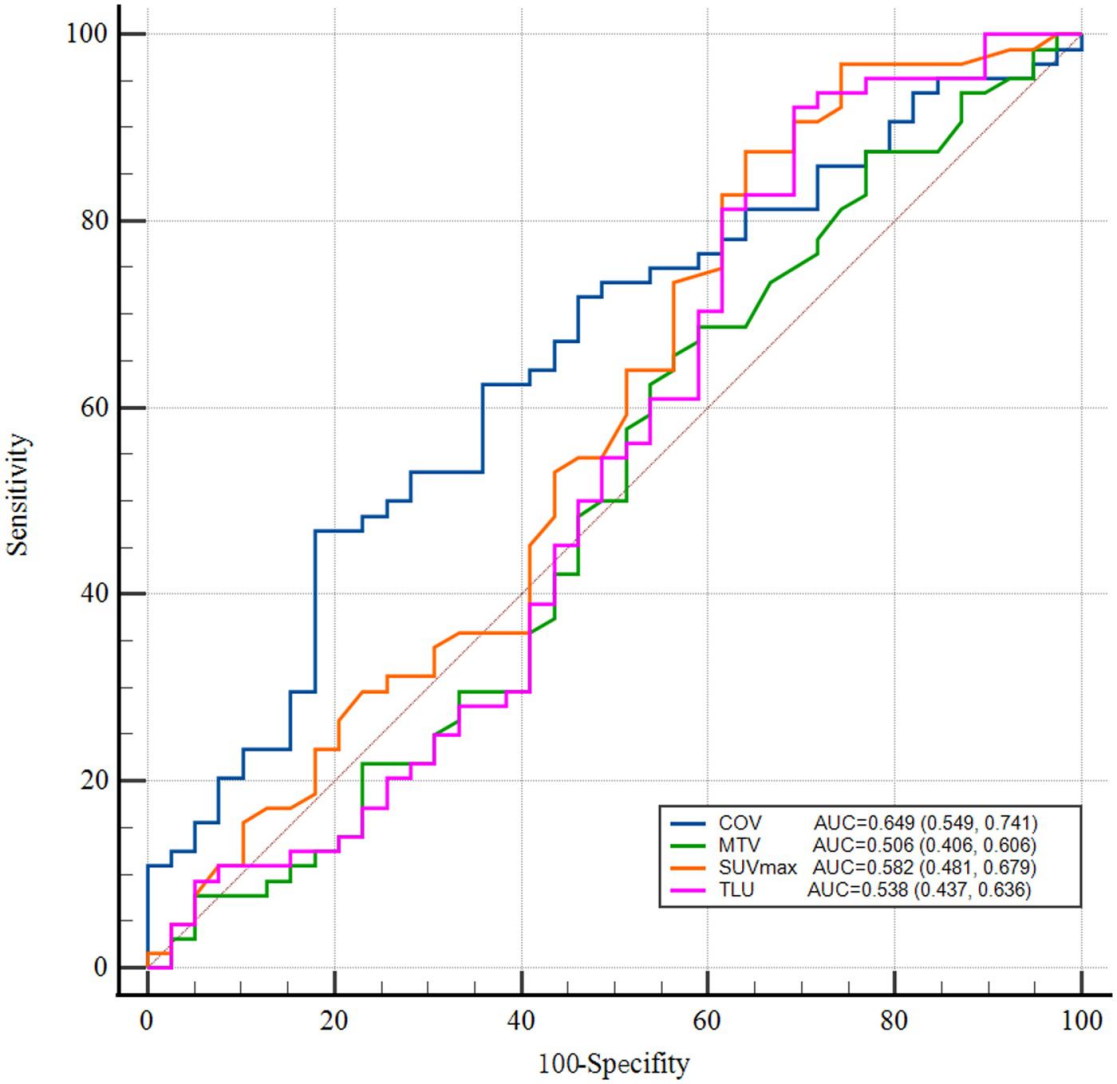
Comparison between COV and other metabolic parameters using ROC curves. The AUC of different metabolic parameters predicting PD-L1 is marked in the lower right corner. Abbreviations: COV = coefficient of variation; MTV = metabolic tumour volume; TLU = total lesion uptake.

According to ROC curve analysis (Figure 3, Table 2), in predicting PD-L1 expression, COV had the best performance (AUC = 0.649), significantly higher than MTV (AUC = 0.506, *P* = 0.0441). Although there was no statistically significant difference between COV and SUVmax or TLU, COV still had the highest AUC. For SUVmax, a cut-off value of 6.3 was proposed, with a sensitivity of 87.5% and specificity of 35.9%. For MTV, a cut-off value of 9.4 was proposed, with a sensitivity of 29.7% and specificity of 59.0%. For TLU, a cut-off value of 6.4 was proposed, with a sensitivity of 92.2% and specificity of 30.8%. For COV, a cut-off value of 28.9 was proposed, with a sensitivity of 46.9% and specificity of 82.1%.

**Table 2.** Evaluation of metabolic tumour parameters for predicting PD-L1 status.

Parameter	AUC (95% CI)	Se (95% CI)	Sp (95% CI)	*P*
SUVmax	0.582 (0.481, 0.679)	0.875 (0.768-0.944)	0.359 (0.212-0.528)	0.2197
MTV	0.506 (0.406, 0.606)	0.297 (0.189-0.424)	0.590 (0.421-0.744)	0.0441*
TLU	0.538 (0.437, 0.636)	0.922 (0.827-0.974)	0.308 (0.170-0.476)	0.0827
COV	0.649 (0.549, 0.741)	0.469 (0.343-0.598)	0.821 (0.665-0.925)	–

Abbreviations: COV = coefficient of variation; MTV = metabolic tumour volume; Se = sensitivity; Sp = specificity; TLU = total lesion uptake.

*P* represents the comparison between the ROC curve constructed by other metabolic parameters and COV.

* *P* < 0.05.

### Identification of prognostic factors for PD-L1 expression

The metabolic parameters were converted from continuous variables to unordered categorical variables based on the cut-off values, and logistic regression analysis was performed together with patient age and gender. The results are shown in Table 3. In the univariate analysis, SUVmax, TLU, and COV were associated with PD-L1 expression (*P* < 0.1), and their odds ratios (ORs) were all greater than 1, indicating that higher values of these 3 metabolic parameters were associated with a higher likelihood of PD-L1 positivity. In further multivariate analysis, COV was identified as the only significant predictive parameter and an independent predictor of PD-L1 expression.

**Table 3.** Univariate and multivariate logistic regression analysis results.

	Univariate analysis			Multivariate analysis		
Parameter	OR	95% CI	*P*	OR	95% CI	*P*
Age	1.034	0.989-1.081	0.144			
Gender	1.876	0.835-4.215	0.127			
SUVmax (<3.7 vs ≥ 3.7)	3.500	1.293-9.476	0.014*	1.007	0.197-5.136	0.994
MTV (<9.4 vs ≥ 9.4 cm^3^)	0.653	0.285-1.496	0.314			
TLU (<6.36 vs ≥ 6.36)	4.636	1.470-14.623	0.009*	3.387	0.579-19.809	0.176
COV (<28.9 vs ≥ 28.9)	3.419	1.364-8.574	0.009*	2.707	1.005-7.287	0.049*

Abbreviations: COV = coefficient of variation; MTV = metabolic tumour volume; TLU = total lesion uptake.

* *P* < 0.05.

## Discussion

ICIs are widely used to treat advanced NSCLC, resulting in prolonged remission. PD-L1 expression is closely linked to higher response rates.^7^ Minimizing patient discomfort can be achieved by replacing PD-L1 status assessment with a non-invasive method, supporting clinical decisions.^8^^,^^9^

Our findings show that the COV of primary tumours detected by 18F-FDG PET/CT is a reliable predictor of PD-L1 expression, outperforming other metabolic markers in diagnostic performance. Previous research has used CT imaging features to predict PD-L1 expression in lung adenocarcinoma. The tumour solidity ratio (consolidation tumor ratio,CTR) correlated significantly with PD-L1 expression (*P* = 0.003), while morphological traits like pleural indentation (*P* = 0.007), spiculation (*P* < 0.01), and ground-glass opacity (*P* < 0.01) were associated with PD-L1 positivity.^10^^,^^11^ However, in different previous studies, the results of feature analysis varied. For example, air bronchogram was found to have predictive value for PD-L1 in one study, while in another study, it was not predictive.

18F-FDG PET/CT provides non-invasive anatomical and metabolic insights into lesions. Takada et al found that SUVmax in NSCLC patients with PD-L1 expression (TPS > 5%) was significantly higher than in those without (*P* < 0.0001). Their multivariate analysis identified high SUVmax as an independent predictor of PD-L1 positivity, with a cut-off of 4.2 offering the best predictive performance.^12^ A study examining PD-L1 expression in lung adenocarcinoma patients found a positive correlation between tumour PD-L1 expression and both SUVmax and TLU (*P* < 0.0001). Additionally, SUVmax was recognized as an independent predictor of PD-L1 expression, with an ideal cut-off value of 9.5.^13^ Unlike our findings, where SUVmax, TLU, and MTV were higher in the PD-L1-positive group, no significant differences were observed in other studies. We speculate that these discrepancies may arise from variations in pathological types and different PD-L1 positivity cut-offs. In our study, PD-L1 positivity was defined as TPS ≥1% in NSCLC patients. Additionally, previous studies did not limit the number of lesions in patients, and in studies assessing treatment efficacy, patients were usually in advanced stages and often had more than one lesion. These differences in threshold definitions and patient inclusion criteria might account for the observed discrepancies in results.

Numerous studies have explored the link between PET/CT imaging radiomics and PD-L1 expression, showing that integrating radiomic features with clinical and pathological factors yields promising results. The AUC for predicting PD-L1 expression >1% was 0.762, and for >50%, it was 0.814.^14^ The selected radiomic features, GLRLM-SRLGE (short run low grey-level emphasis-Short Run Low Gray Level Emphasis) and NGTDM-coarseness（Neighbouring Gray Tone Difference Matrix), are linked to metabolic heterogeneity. They capture details such as the direction, spacing, and magnitude of greyscale changes within lesions, reflecting their complexity.^15^^,^^16^ To supply the oxygen needed for tumour growth, numerous new blood vessels form within the tumour, resulting in irregular blood flow and perfusion. The presence of these blood vessels and necrotic tissue contributes to increased texture heterogeneity.^17^^,^^18^

In the study by Zhao et al, it was found that tumour regions with high SUV(max) expressed a higher proportion of hypoxia-related marker hypoxia-inducible factor-1α (63.1% vs 37.9%, *P* = 0.024) compared to tumour regions with low SUVmax. The central region of the tumour had higher HIF-1α, corresponding to higher SUVmax, indicating the correlation between FDG uneven distribution and hypoxia.^19^ van Baardwijk et al performed 18-F FDG PET/CT scans on postoperative specimens of NSCLC patients and used SUVmax of 80%, 50%, and 20% as thresholds to delineate regions with different uptake levels in lesions. Pathological examinations were conducted on different regions, confirming that the differences in tumour 18-F FDG uptake imaging were related to tissue composition. Regions with low uptake had more fibrous components, while regions with high uptake had more tumour components. This suggests that analysing the heterogeneous distribution of 18-F FDG uptake described in PET images can reflect tumour heterogeneity.^20^

To our knowledge, no studies have explored the relationship between PD-L1 expression and metabolic heterogeneity in PET images. Previous research has used PET-based metabolic heterogeneity measures to predict EGFR mutations in NSCLC, as EGFR is a key regulator of PD-L1 expression.^21^ Of the different heterogeneity indices, 1/COV was found to be a significant predictor of EGFR mutation status (*P* < 0.02). This measure is independent of tumour metabolic volume, relying instead on the lesion’s mean uptake and the SD of uptake, representing the internal variability within the lesion.^22^ Studies by Li et al^23^ and Pellegrino et al made the following research.^24^ Metabolic heterogeneity was significantly associated with pathological classification, differentiation degree, and T stage in NSCLC (*F* = 17.611, 23.932, 11.082, all *P* < 0.001). As tumour differentiation decreased and T stage increased, metabolic heterogeneity also increased. COV emerged as a potential predictor for prognosis in late-stage NSCLC. Kaplan–Meier analysis showed that patients with lymph node COV ≤0.29 had improved overall survival (OS) compared to those with COV >0.29 (*P* = 0.0147). Additionally, the PD-L1-positive group exhibited a significantly higher COV than the negative group (*P* = 0.011), with COV acting as an independent predictor of PD-L1 expression (OR = 2.707, *P* = 0.049), indicating that patients with higher lesion metabolic heterogeneity have better effects on targeted therapy. Although extreme values appeared in the box plot, the statistical method for COV was rank-sum test, and COV was converted from quantitative data to binary data based on a cut-off, which greatly minimized the influence of extreme values on the results.

In a meta-analysis that included 7 studies, the authors observed that 18F-FDG PET/CT demonstrated moderate sensitivity and specificity in predicting PD-L1 expression in solid tumours. The pooled sensitivity, without heterogeneity, was 0.75 (95% CI: 0.65-0.82), while the pooled specificity, with heterogeneity, was 0.73 (95% CI: 0.64-0.81).^25^^,^^26^ This is consistent with our study results. Choosing 28.9 as the COV cut-off, the sensitivity was only 46.9%, but the specificity reached 82.1%, indicating that COV can accurately identify PD-L1 negative cases. Given the goal of this study to identify patients who are more likely to benefit from immune-targeted therapy, the high specificity of the COV is useful for selecting PD-L1-negative patients, making it more applicable for guiding targeted treatment.

Our study has several limitations. First, as a single-centre study with a small sample size, its findings may not be broadly generalizable. Second, being retrospective, it included patients who underwent 18F-FDG PET/CT after CT or haematological screenings identified suspicious lesions, introducing potential selection bias. Future prospective, multicentre studies with larger sample sizes are needed to validate these results. Additionally, as no previous studies have explored the relationship between COV and PD-L1 expression in NSCLC, we focused on patients with a single lung lesion to reduce complexity. Further research should address lesion heterogeneity. Finally, our study did not analyse adenocarcinoma and squamous carcinoma separately, which will be explored in future research. Our study demonstrated that 18F-FDG PET/CT metabolic parameters can effectively predict PD-L1 expression in NSCLC patients, with COV being the most accurate predictor of metabolic heterogeneity. Higher COV values were associated with positive PD-L1 expression. Incorporating COV data from primary tumours into clinical decision-making could provide valuable insights for selecting targeted therapies, ultimately improving patient outcomes.
